# Role of Chronobiology as a Transdisciplinary Field of Research: Its Applications in Treating Mood Disorders

**DOI:** 10.4274/balkanmedj.2017.1280

**Published:** 2017-12-01

**Authors:** Okan Çalıyurt

**Affiliations:** 1 Department of Psychiatry, Trakya University School of Medicine, Edirne, Turkey

**Keywords:** Chronotherapy, circadian rhythm, diagnosis, mood disorders, phototherapy, biological clocks

## Abstract

Chronobiology is a field that studies the effects of time on biological systems. Periodicity is of particular interest. The master biological clock in the suprachiasmatic nucleus controls daily rhythms of core body temperature, rest-activity cycle, physiological and behavioral functions, psychomotor functions and mood in humans. The clock genes are involved in the generation of the circadian rhythms and the biological clock is synchronized to solar day by direct photic inputs. Various circadian rhythm abnormalities have been demonstrated in mood disorders such as unipolar depression, bipolar depression and seasonal affective disorder. Hypotheses involving circadian rhythm abnormalities related to the etiology of mood disorders have been raised. The resulting circadian rhythm changes can be measured and evaluated that these techniques can be used to identify subtypes of mood disorders associated with circadian rhythm changes. The data obtained from chronobiological studies reveal methods that manipulate circadian rhythms. The effects of light and melatonin on circadian rhythms are determined by these studies. Chronobiological research has been applied to the psychiatric clinic and light therapy has been used as a chronotherapeutic in the treatment of mood disorders. On the other hand, chronotherapeutic approaches with effects on circadian rhythms such as sleep deprivation therapy have been used in the treatment of mood disorders too. As a good example of translational psychiatry, chronobiological studies have been projected in the psychiatry clinic. It may be possible, the data obtained from the basic sciences are used in the diagnosis of mood disorders and in the treatment of psychiatric disorders as chronotherapeutic techniques. Developments in the field of chronobiology and data obtained from chronotherapeutics may enable the development of evidence-based diagnosis and treatment in psychiatry.

Chronobiology is the study of biological rhythms. It examines the effects of time on biological events and internal biological clocks. Over the past few decades, chronobiology has developed into a multidisciplinary field of interest in general medicine and psychiatry (1). Research on chronobiology is constantly growing and has contributed to the development of an interdisciplinary field of research. The Nobel Prize in Medicine in 2017 was awarded to scientists who investigated molecular mechanisms that control circadian rhythms.

The earth’s rotation and the daily light-dark or day-night cycle have had long and constant biological impacts on the earth’s living organisms. Over the long evolutionary process, organisms have developed clock-like structures. These biological clocks help organisms successfully perform their activities over a 24-h cycle. This adaptive ability predicts cyclic changes and allows behavioral and physiological harmonization. These endogenous clocks are useful because they can work independently and keep time without requiring time clues from external light-dark changes (2).

The master circadian clock is located in the suprachiasmatic nucleus (SCN) of the anterior hypothalamus. The SCN controls many circadian rhythms such as the sleep-wake cycle, core body temperature (CBT), mood, and psychomotor performance. The circadian control of homeostatic functions occurs through neurons showing approximately hours of oscillation (3). The SCN needs daily synchronization to adapt to environmental 24- light-dark cycles, and light is the major synchronizer (zeitgeber). The SCN receives light information through the retinohypothalamic pathway. This process is mediated by ganglion cells in the retina. The SCN can be synchronized with nonphotic stimuli. Social interactions, daily routines, meal timing, and physical exercise are other synchronizers. The SCN receives feedback from the pineal gland. Circadian rhythms are found throughout the cells of the body, and the SCN allows them to synchronize as a primary oscillator (4).

The physiological and behavioral functions of organisms are under circadian control. Any disruption occurring in circadian rhythms can affect biological and mental processes. As a result, such disruptions can lead to the development of physical or mental disorders (5). Circadian rhythm disruptions are also seen in many psychiatric disorders, particularly mood disorders (6).

## Chronobiological Aspects of Mood Disorders

The present article continues to use the term “mood disorders” rather than the new classification in the Diagnostic and Statistical Manual of Mental Disorders (DSM)-5 to ensure easy understanding. Mood disorders are among the most common mental disorders. In a large European study, the prevalence of lifetime occurrences of any mood disorder was reported to be 14% (7). Major depression is the most common and a serious mood disorder. If it is not effectively treated, it can have serious consequences. The etiology of mood disorders may involve neurotransmitter changes, neuroendocrine and neuroimmune regulation or brain structure abnormalities, or genetic and psychosocial factors. While the etiology of mood disorders is not fully understood, it is known to be multifactorial and complex. Various chronobiological changes have been observed in mood disorders. These changes may either be a result of mood disorders or a part of the clinical picture. Chronobiological features observed in mood disorders may be involved in the etiology of mental disorders (8).

Circadian rhythms are affected during depression. Among the major circadian and sleep abnormalities related to depression are diurnal variation in mood, increased mean core temperature and decreased period amplitude, phase advance of cortisol and monoamines, abnormal melatonin secretion, insomnia, sleep disturbances, shortened REM sleep latency, and early morning awakening (9). Circadian rhythm abnormalities are seen in acute and euthymic periods of bipolar disorder.

The sleep-wake cycle is the most important circadian rhythm. Sleep disorders in depressed patients are common and are among the criteria for diagnosing mood disorders. They are also common in bipolar disorder and may precede the onset of mania and depression. During a manic episode, the need for sleep is reduced, and during the euthymic period, most patients experience sleep disturbances (10).

Seasonal affective disorder (SAD) is a type of depression that has been associated with desynchronization between circadian rhythms and light-dark cycles during short photoperiod seasons. SAD is associated with hypersomnia and delayed circadian rhythms (11). The pathogenesis of SAD is thought to involve melatonin, which is a hormone associated with circadian rhythms (12).

The levels of neurotransmitters, such as serotonin, norepinephrine, and dopamine, known to be involved in the development, course, and treatment of mood disorders show circadian rhythms. Similarly, their receptors are controlled by circadian rhythms (13,14). This neuronal chemical activity is regulated by the expression of clock genes and is controlled by various feedback mechanisms (15). In healthy individuals, a relationship has been found between the evening type in the Morningness-Eveningness Questionnaire and depressive symptoms. Similarly, bipolar depression has been associated with evening preference (16). An increased frequency of depression occurs in circadian rhythm sleep disorders such as advanced sleep phase syndrome, delayed sleep phase syndrome, and shift work (17,18). Depressive symptoms have also been shown to be associated with jet lag, which leads to a disruption in circadian rhythms (19).

Biological clock and circadian rhythms are useful if they can be adjusted to the local time, allowing one’s alertness level to be set during the day. The subjective mood of a person changes with the circadian phase during the day. The duration of prior wakefulness also affects mood (20). This relationship is bidirectional, and mood can affect biological rhythms. As disruptions in the circadian timing system can be related to mood disorders, failure of the biological clock to function properly, damage to the clock mechanism, or the presence of a genetic defect may be involved in the etiology of mood disorders. A significant relationship appears to exist between the regulation of mood and the functionality of the circadian system. However, this relationship has not been proven and continues to be a matter of debate (6).

Chronobiological features associated with mood disorders are important in clinical practice. As noted above, many circadian disruptions have been reported with mood disorders. Features associated with mood disorders can shed light on the etiology and diagnosis of unipolar depression, bipolar disorder, and SAD.

## Chronobiological Basis for Diagnosing Mood Disorders

Diagnostic systems widely used in the field of psychiatry merely form a common communication tool. Diagnostic classification systems, such as the DSM and the International Classification of Diseases, unfortunately allow only a diagnosis based on symptoms and signs (21). Moreover, most of their current diagnostic categories of classification systems have not been validated yet. High comorbidity rates have been a controversial consequence of these classification systems (22). Thus, these diagnostic systems, which do not rely on the underlying etiology and pathophysiology of diseases, have many limitations. Their most important impact is related to the treatment of psychiatric disorders. As the underlying causes of psychiatric disorders are not fully understood, etiology-based treatment cannot be developed.

An alternative to these diagnostic systems is the Research Domain Criteria (RDoC) project. The RDoC introduces a translational approach (23). It aims to use underlying mechanisms to diagnose disorders based on neuroscientific research. The diagnostic use of data obtained from chronobiological studies for treating mood disorders could thus be an alternative approach in psychiatric diagnosis.

The relationship between chronobiological changes and mood disorders is still unclear. Most of our behavioral and physiological functions show circadian rhythmicity, and at the heart of this is the biological clock. Because of this, biological or mental problems may arise with the disruption of this circadian timing system. Similarly, mood disorders are more common when genetically disordered circadian anomalies are present. The incidence of depression is increased in delayed or advanced sleep phase circadian rhythm sleep disorders. Genetic variations have been shown between sleep disorders and circadian genes (24).

Various hypotheses have been stated to explain chronobiological changes in mood disorders and the etiology of these disorders. In depression, a decrease in REM sleep latency, early morning awakening, and a shift in the release of melatonin indicate that circadian rhythm phases advance in relation to the sleep-wake rhythm. Other hypotheses include homeostatic and circadian process-related disturbances, phase delay, amplitude diminution, and impairment in social rhythms (9,25).

Circadian rhythms are also observed at the monoamine neurotransmitter level and in the release or synthesis of these neurotransmitters, which are involved in the etiology of depression and mood regulation. Similarly, their receptors operate according to circadian rhythm (26). However, it is still unclear wow circadian control is achieved. Changes in circadian rhythms that may occur in this system directly affect mood. The evaluation of neurotransmitters and their receptors involved in mood regulation or the course of mood disorders within diagnostic processes has led to new insights. It would be more appropriate to evaluate them not only quantitatively but also in terms of circadian changes. Thus, there may be an opportunity to make a proper diagnosis of the subtype of a mood disorder caused by circadian factors. This etiology-based diagnosis will provide the grounds for etiology-based treatment.

If observed circadian rhythm changes in some psychiatric disorders, particularly mood disorders, can be measured in clinical practice, it will be possible to obtain data that can be used for diagnosing these disorders. Studies in animals and humans on chronobiology have brought the problem of the measurement of circadian rhythms to certain standards. These measurements allow the phase, period, and amplitude data of circadian rhythms to be evaluated (27). A translational approach should be used at this point, and studies on chronobiology can be transferred to psychiatric clinics. We have the opportunity to obtain objective data when diagnosing psychiatric disorders, particularly disorders corresponding to circadian hypotheses.

There are many methods of measuring circadian rhythms in humans. These methods ensure that at least one cycle is recorded by taking measurements for longer than 24 h.

A reliable instrument for measuring circadian rhythms is CBT. CBT provides long-term, continuous, and inexpensive measurements (28). Melatonin and cortisol circadian rhythms are other reliable indicators of the biological clock. The ability to evaluate melatonin levels in saliva, as well as in bllood, makes it an easy-to-use instrument. Melatonin levels that are not influenced by factors such as sleep and posture have a significant advantage over CBT. Dim light melatonin onset is also used as a reliable alternative measurement tool for circadian rhythms (29).

The measurement of circadian rhythms has limitations. Some natural factors have masking effects on circadian rhythms. For example, nighttime light exposure can affect circadian rhythms by stimulating activity and inhibiting melatonin secretion. Sleep episodes also affect circadian rhythms. Although the sleep-wake cycle is a major circadian rhythm and is controlled by circadian and homeostatic processes within the two-process model, sleep period has a masking effect on circadian rhythms. In addition, posture, light intensity, and activity level changes that occur in the sleep episode lead to decreases in CBT. Therefore, the elimination of masking factors using special techniques is important for the accurate measurement of circadian rhythms.

One of the most important of these techniques is imposing a consistent routine by removing or keeping all masking factors constant. In this protocol, individuals are kept in an isolated environment. A full circadian cycle is recorded, with a measurement period of at least 24 h. Individuals remain close to the supine position, and social communication is restricted. Environmental light and room temperatures are kept constant. The administration of food and beverages is equally divided throughout the measurement period and they are given as hourly isocaloric snacks. All other factors, such as noise and smell, are removed. Individuals are kept awake during the measurements; thus, the masking effects of sleep are removed. As a result, the constant routine protocol helps provide reliable and valid information on circadian rhythms (30).

The forced desynchrony protocol is another method used to study circadian rhythms. Individuals stay in an isolated environment in which sleep-wake times deviate from 24 h. This removes the masking effect of the sleeping period on the circadian rhythms. This method can reveal the relationship between circadian and homeostatic processes. Twenty or twenty-eight-hour sleep-wake cycles are preferred. The protocol is performed in dim light conditions, eliminating the masking effect of light on circadian rhythms (27).

Questionnaires such as the Horne and Ostberg Morningness-Eveningness Questionnaire and the Munich Chronotype Questionnaire or actigraphic methods provide noninvasive information on circadian rhythms (31,32). A recent meta-analysis revealed an association between depressive symptomatology and individual chronotypes. In particular, night-oriented chronotypes revealed increased symptoms of depression and mood disorders (33). Actigraphy provides objective and inexpensively obtained data on circadian rhythms by taking recordings of sleep-wake cycles over several weeks (34).

Some circadian rhythm genes have been shown to be involved in the development of mood disorders (24). Polymorphisms in various circadian rhythm-related genes in mood disorders and SAD have revealed a genetic relationship between them (35,36). In addition, a relationship has been reported between epigenetic mechanisms and circadian rhythm regulation (37). Therefore, genetic tests and assessments may be of value in the diagnosis of psychiatric disorders, particularly mood disorders. It may also soon be possible to use genetic testing to detect genetic changes in the circadian clock in patients with mood disorders. Similarly, screening genetic polymorphisms will allow subtypes associated with mood disorders to be identified. The identification of possible genetic markers will ensure making a specific diagnosis.

Techniques have been developed to measure and evaluate circadian rhythm changes accompanying mood disturbances. Data obtained can be used to objectively diagnose and monitor mood disorders. In the near future, genetic tests may serve diagnostic functions for treating mood disorders. Biological parameters compatible with etiological mechanisms can be used instead of descriptive methods for treating mood disorders.

## Treatment of Mood Disorders in Light of Chronobiological Developments

We can identify chronobiological changes underlying some psychiatric disorders and use them as diagnostic tools. However, developments are reflected in treatment applications as well. Drugs used for the treatment of various diseases need to be administered according to circadian rhythms. Even the benefits and side effects of treatments related to the weekly, monthly, seasonal or annual biological rhythms of individuals must be related (38). Most successful therapeutic approaches for the treatment of mood disorders impact circadian rhythms. These effects can take the form of resetting, shifting, or stabilizing rhythms. In addition, most individuals with mood disorders benefit from the strict regulation of sleep-wake hours (39). Chronobiological investigations have demonstrated the effects of light on circadian rhythms and have deemed it as a therapeutic tool. In addition, the effects of sleep deprivation and melatonin on circadian rhythms have been demonstrated.

**FIG. 1.**
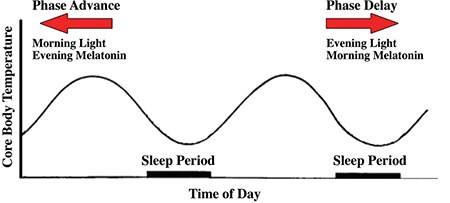
Phase-shifting effects of light and melatonin.

Bright light therapy: Light is the dominant zeitgeber for humans and can induce phase shifts in circadian rhythms. The effect of light on circadian rhythms is related to certain parameters. The time of day when exposed to light and the intensity of light determine the effects. Exposure to light at night causes phase delay, while exposure in the morning causes phase advance in circadian rhythms (Figure 1) (25). Along with the intensity of light, the duration of exposure and the number of days of therapy play roles in determining its effect. Even the wavelength of applied light is important. Studies have shown that blue light is most effective in phase-shifting circadian rhythms (40).

Light therapy is beginning to be used to treat SAD (11) and has become the first-line treatment for SAD (41). It then came to be used for treating other mood disorders and psychiatric disorders. Light therapy has also been proven effective in patients with nonseasonal depression and bipolar depression (42). Light therapy may also have applications in various other diseases such as perinatal depression, premenstrual dysphoric disorder, Parkinson’s disease, Alzheimer’s disease, circadian rhythm sleep disorders, and schizophrenia (Table 1) (41).

Light therapy is provided by light therapy devices. These devices are also called light boxes. They usually contain fluorescent lamps covering the entire light spectrum. Ultraviolet filters are used to increase safety. In clinical practice, a popular 10.000-lux lightbox is kept at the level of the eyes for 30 min. This serves to stimulate the retina via a light source (43). Apart from light boxes, portable light visors that can be mounted on the head and dawn-simulator devices have also been developed (44).

Along with its therapeutic benefits, the safety of light therapy is an advantage. It is well tolerated by most patients, and its side effects, which include nausea, headache, and agitation, are mild and transient (41).

Sleep deprivation therapy (wake therapy): Sleep deprivation therapy is an alternative treatment that impacts circadian rhythms and the biological clock. It is a chronotherapeutic technique used for mood disorders, particularly in unipolar and bipolar depression patients. Sleep-wakefulness is regulated by two interacting biological processes: process C (circadian) and process S (homeostatic). Hypotheses related to the effect of sleep deprivation therapy on homeostatic and circadian processes have been reported (45,46,47,48). Sleep deprivation therapy is one of the fastest-acting treatments for depression. Totally, 60% of patients show an antidepressive response after a single administration; responses are even more in bipolar depressive patients (45).

**FIG. 2.**
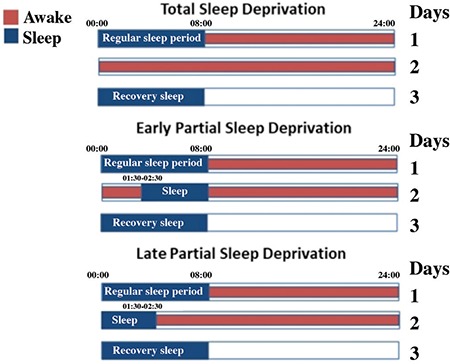
Schematic presentation of total and partial sleep deprivation. Each horizontal bar represents one day. Red areas indicate the time period in which sleep deprivation is applied.

In clinical practice, sleep deprivation encompasses an entire night and the following day. Patients go to sleep the next day at their usual bedtime. Variations in sleep deprivation therapy are used in clinical practice. These are mainly partial sleep deprivation practices. They are administered as early or late sleep period deprivation. Late partial sleep deprivation is an alternative to total sleep deprivation because of its effectiveness, high patient compliance, and low side effects (43) (Figure 2).

Melatonin: Melatonin is secreted from the pineal gland, and it enables the transmission of photoperiodic information. With the healthy functioning of melatonin, all tissues in the body can access information about the time of day and year. The pineal gland is controlled by the circadian system. However, the relationship is two-sided, and darkness information that reaches the pineal gland also affects circadian rhythms. Melatonin functions as an important zeitgeber, and melatonin has sleepiness-promoting properties. Because of these properties, melatonin is used as a drug for treating disorders in which circadian rhythms are affected (49).

Melatonin, which is used for treating circadian rhythm sleep disorders such as jet lag or shift work, may also play a potential role for treating other psychiatric and mood disorders in which circadian rhythms are disturbed (50). The effects of melatonin on depression are controversial. However, changes in melatonin levels in mood disorders have been reported in research, and newly developed melatonergic drugs have been proved to be successful in treating major depression (51,52). In addition, melatonin can be successfully used to synchronize the circadian rhythms of blind individuals (49). Hence, melatonin and melatonergic drugs may take place as an alternative in treating mood disorders. Melatonin can be administered as a pharmacological agent. It is available in various doses in the market. The significance of its clinical use as a chronotherapeutic is that its effects on circadian rhythms are exactly the opposite as those of light therapy (Figure 1).

**FIG. 3.**
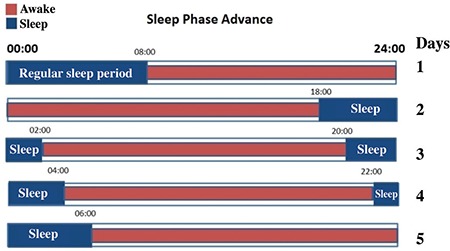
Schematic presentation of sleep phase advance. Each horizontal bar represents one day. Red areas indicate the period of stay awake.

Sleep phase advance: The phase-advancing sleep-wake cycle is another chronotherapeutic approach for treating mood disorders (53). In this technique, sleep-wakefulness is advanced 6 h. In the second half of the night, the patient is kept awake. Then, sleep-wake cycles are gradually brought to the usual times. In this process, antidepressant activity can be achieved within a few days in depressive patients (Figure 3) (43).

The use of chronotherapeutic approaches in the field of psychiatry is still under development. Combinations of chronotherapeutics and drug treatments can be used to increase treatment success. Similarly, chronotherapeutics can be used in combination (54). Rapid antidepressant responses have been reported from the combination of three different chronotherapeutics, such as sleep deprivation therapy, light therapy, and sleep phase advance (55). Finally, a positive response to some chronotherapeutics was associated with a favorable response to others (56).

Thus, the data obtained from chronobiological studies can be used to develop treatment tools for psychiatric disorders. The use of light therapy for mood disorders is the best example. The known effects of light on circadian rhythms can be successfully used as a clinical treatment. In addition, chronotherapeutics such as sleep-wake manipulation, sleep deprivation therapy, sleep phase advance, or exogenous melatonin can be used in treating mood disorders. All these chronotherapeutics act on circadian rhythms.

Chronobiology is a continuously evolving field, and developments in this area can result in new implications for human health and disease. Chronobiology impacts not only biology but also fields such as medicine, pharmacology, and psychology. Translational research in chronobiology can provide insights into the diagnostic and therapeutic processes of mood disorders in the field of psychiatry. Previous research has revealed a complex relationship among circadian rhythms, mood, and behavior, and translational research can help clarify the relationship (57,58,59,60,61).

Chronobiological research can also contribute to the understanding of the causes of circadian changes and the effects of chronotherapeutic techniques in mood disorders. Chronobiological studies, such as translational research in the field of psychiatry, may bring about developments in the diagnosis and treatment of mood disorders in the future. In addition, chronobiological and chronopharmacological investigations may contribute to the development of antidepressants and mood stabilizers with specific effects on circadian rhythms. In addition, nondrug-based chronotherapeutic techniques can have applications for conditions where medications cannot be used, such as advanced liver and kidney failure or mood disorders in the peripartum period. Therefore, chronobiological developments may help overcome the problem of drug-dependent approaches for treating mood disorders.

The data obtained on current chronotherapeutic approaches may also be a source for future chronobiological investigations. Such research could provide new data on the etiology and diagnosis of mood disorders. With this, the connection between chronobiology and chronotherapy can be more strongly established in the field of psychiatry.

**Editor-in-Chief's note:** Okan Çalıyurt is the member of the Editorial Board of Balkan Medical Journal. However, he did not take place at any stage on the editorial decision of the manuscript.

## Figures and Tables

**Table 1:**
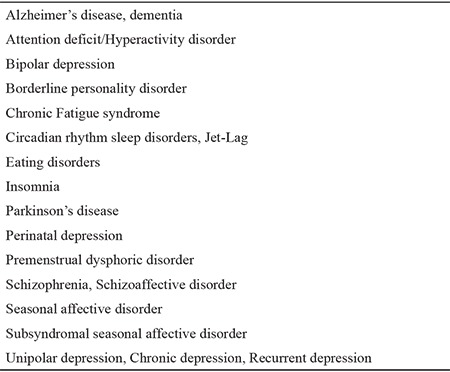
Current and emerging indications of light therapy
